# Extensively Hydrolyzed Formula Improves Allergic Symptoms in the Short Term in Infants with Suspected Cow’s Milk Protein Allergy

**DOI:** 10.3390/nu15071677

**Published:** 2023-03-30

**Authors:** Michael J. Wilsey, Jared Florio, Jesse Beacker, Luke Lamos, Jessica V. Baran, Lea Oliveros, Panida Sriaroon, Jerry M. Brown, Jon A. Vanderhoof

**Affiliations:** 1 Department of Pediatrics, University of South Florida Morsani College of Medicine, Tampa, FL 33602, USA; 2 Pediatric Gastroenterology, Hepatology, and Nutrition of Florida, St. Petersburg, FL 33701, USA; 3 Office of Medical Education, Florida Atlantic University Charles E. Schmidt College of Medicine, Boca Raton, FL 33431, USA; 4 Department of Pediatric Allergy and Immunology, University of South Florida Morsani College of Medicine, Tampa, FL 33602, USA; 5 Department of Gastroenterology Hepatology and Nutrition, Boys Town Hospital, Boys Town, NE 68010, USA

**Keywords:** cow’s milk protein allergy, extensively hydrolyzed formula (eHF), food allergy, Lactobacillus rhamnosus (LGG), zsmoments

## Abstract

Although extensively hydrolyzed formula is widely accepted for managing cow’s milk protein allergy (CMPA) long-term, there is a lack of evidence on its short-term efficacy. This study’s objective was to investigate the short-term symptom changes (within 3–6 weeks) of infants diagnosed with CMPA and managed with extensively hydrolyzed formula containing Lactobacillus *rhamnosus* at their subsequent physician visit. Healthcare providers treating 202 patients diagnosed with CMPA under six months old completed de-identified surveys, which were then analyzed in this prospective study. After their first visit, the patients were started on extensively hydrolyzed formula, and their baseline symptoms were scored on a severity scale of 0–3. Patients were then reevaluated at their next follow-up visit to assess changes in symptom severity. The study found statistically significant improvements in gastrointestinal (93%), skin (83%), respiratory (73%), and uncategorized symptoms (90%). These symptom improvements were consistent across different follow-up visit durations. This study is the largest prospective analysis conducted in the United States evaluating short-term change in CMPA symptoms severity in infants under six months old using extensively hydrolyzed formula. These findings suggest that extensively hydrolyzed formula is associated with clinical symptom relief, which is often noticeable by the next follow-up visit. However, additional randomized control trials are needed to validate these results.

## 1. Introduction

Cow’s milk protein is a major food allergen in young children, affecting an estimated 2–3 percent of children [1]. Cow’s milk protein allergy (CMPA) occurs commonly during infancy, with the highest prevalence in the first year of life, and affects infants fed with cow’s milk-based formula as well as those who are exclusively breastfed [2,3]. CMPA can be classified into two distinct types based on the mechanism of reaction and onset of symptoms: Immunoglobulin-E (IgE) mediated (immediate hypersensitivity) and non-IgE mediated (cell-mediated or delayed-type hypersensitivity) [4]. IgE-mediated symptoms occur within 30 min after ingestion of cow’s milk protein, while non-IgE-mediated symptoms occur hours to days after ingestion of cow’s milk protein [5]. CMPA generally affects the skin, respiratory, and digestive systems and encompasses specific symptoms such as urticaria, angioedema, wheezing, chronic cough, vomiting, diarrhea, colic, rectal bleeding, and complications such as chronic diarrhea, iron deficiency anemia, GERD, and chronic vomiting [1,2,6]. Symptoms can vary greatly in severity and frequency based on the mechanism of CMPA as well as the location of the GI tract affected. Unfortunately, there are no current pathognomonic symptoms of CMPA, which increases the difficulty of a definitive diagnosis. Diagnosis is further complicated by the need to differentiate CMPA from other allergies to cow’s milk [7]. An elimination diet is the gold standard diagnostic measure, which involves removing cow’s milk protein from both the mother’s and infant’s diet for 3–5 days if immediate reactions of CMPA are present, and 2–3 weeks if delayed symptoms are present [1,8]. An oral challenge test in which the reintroduction of cow’s milk protein causes the recurrence of the same symptoms can also be helpful [1,9].

Treatment for CMPA includes an elimination diet of 2–4 weeks that involves removing cow’s milk protein from the mother’s diet if breastfeeding (first-line treatment), as well as usage of hypoallergenic formula [1]. The two hypoallergenic formulas of choice are extensively hydrolyzed formula and amino acid-based formula (AAF), which have well-established evidence of having long-term efficacy in CMPA treatment [10,11,12]. Hypoallergenic formula usage is further encouraged when CMPA is suspected in non-breastfed infants, with extensively hydrolyzed formulas being the first-line formula of choice [1]. Extensively hydrolyzed formula with Lactobacillus rhamnosus GG (LGG), a probiotic, specifically has been shown to be not only effective in the long-term treatment of CMPA, but also in accelerating the tolerance acquisition in children with CMPA when compared with AAF, soy formula, and hydrolyzed rice formula (RHF) [13]. Tolerance acquisition in CMPA is a subject that has been increasingly researched in recent years due to shifts from passive to proactive interventions for better tolerance outcomes in patients [14]. Another study comparing extensively hydrolyzed formula with and without LGG found that extensively hydrolyzed formula with LGG specifically leads to a decreased incidence of allergic manifestations and an increased rate of oral tolerance in children with IgE-mediated CMPA [15]. Lastly, the safety and efficacy of extensively hydrolyzed formula compared to other treatment formulas such as has been found to lead to decreased incidence of CMPA-related symptoms and better tolerance long-term [16]. However, data regarding the short-term usage and efficacy of extensively hydrolyzed formula with LGG for CMPA, which is a major goal of care for both healthcare professionals (HCPs) and parents, are limited [17].

The primary goal of this study is to assess the short-term change of allergic symptoms in infants under six months of age with symptoms consistent with CMPA treated with a commercial extensively hydrolyzed formula containing LGG. Our study proposes that using eHF to manage CMPA in infants will result in notable short-term improvements in symptoms. Specifically, we hypothesize that infants diagnosed with CMPA by HCPs and treated with eHF will exhibit reduced symptom severity during their next physician visit.

## 2. Materials and Methods

### 2.1. Study Design and Participants

HCP inclusion criteria for this study included >2 years of clinical experience in an office-based setting; a primary specialty in General Pediatrics, Pediatric Allergy/Immunology (A/I), Pediatric Gastroenterology (GI), or General Gastroenterology; and seeing at least two newly diagnosed CMA patients per week. HCP exclusion included non-submission of visit 2 information for any of the patients involved in the original study population. Demographic data were also collected for all HCPs involved in the treatment of the study’s patients that included gender, specialty, years in practice, clinical practice setting, and the average number of CMPA patients weekly.

This study is a prospective cohort analysis of de-identified survey data that were collected from HCPs that analyzed the clinical profile of infants that displayed symptoms consistent with CMPA or that were diagnosed with CMPA between July 2021 to October 2021. Infants under the age of 6 months with symptoms consistent with CMPA or diagnosed with CMPA via clinical assessment (by a pediatrician and/or pediatric gastroenterologist) were included in the study. Inclusion criteria included infants prescribed with eHF LGG at the first enrollment visit (Visit 1) and patients with survey data collected by their HCP at the time of treatment initiation and then at the follow-up visit (Visit 2). Exclusion criteria included infants that did not receive eHF LGG as their treatment formula at Visit 1, infants > 6 months of age at the time of starting their treatment formula, patients that did not have their survey data collected at the time of treatment initiation (Visit 1) and four weeks after treatment at Visit 2, and any patients that either discontinued or switched treatment between Visit 1 and Visit 2. The local Institutional Review Board approved this study on 28 April 2021 (IRB00279920).

### 2.2. Data Collection

Data were collected for the study at Visit 1 and at Visit 2. The HCPs recorded all data on ZSMoments, a mobile-based platform by ZS Associates that allows for rapid, secure data collection on a user’s mobile device. All patient data were de-identified (name, birth dates), with no possible identifiers being recorded. For each patient, the HCP filled out the 10 min ZSMoments survey to assess and document the baseline symptoms of CMPA at Visit 1 and at Visit 2. Demographic data collected included patient characteristics (age, gender, and height/weight percentiles), and family history of allergies.

At Visit 1, the patient’s CMPA symptoms were scored by the assessing HCP on a severity scale of 0 to 3. The scale parameters were the following: 0 representing “symptom not present”; 1 representing the severity of “mild”; 2 representing the severity of “moderate”; and 3 representing the severity of “severe.” Each patient was evaluated on the methods used to diagnose their CMPA, which included clinical assessment, family history, elimination diet, food panel testing, food antigen re-challenge, skin prick testing, serum IgE assay, and atopic patch testing. Patient management for CMPA at the time of Visit 1, including the usage of antihistamines, probiotics, elimination diet while breastfeeding, and usage of any hypoallergenic formula, was also evaluated.

Patients were then started on eHF LGG as their treatment formula. At Visit 2, data collected included patient assessment for any changes in symptom severity, repetition of symptom severity scoring, and determining changes in CMPA symptoms. Data collected at Visit 2 also included the following: the date of the patient’s Visit 1, the method used to diagnose the patient’s CMPA, the number of days since the patient’s Visit 1, and the patient’s current height and weight percentiles.

After data collection at Visit 2 for each patient was acquired, the symptom severity scale was utilized to compare symptom changes between Visit 1 and Visit 2. Each patient will have an aggregated score based on the severity scale for the four categories of symptoms: gastrointestinal (GI), skin, respiratory, and other. The scale number for each symptom category will be calculated and added together to form the aggregated score. This will be done with the symptom severity numbers at both Visit 1 and Visit 2. Symptom improvement will be defined as a reduction in the aggregate symptom score between the Visit 1 and Visit 2; for example, an aggregate number of 6 (Visit 1) compared to 2 (Visit 2). Symptom worsening will be defined as an increase in the aggregate symptom score between Visit 1 and Visit 2 for example, an aggregate number of 2 (Visit 1) compared to 5 (Visit 2). No improvement will be represented by no change between Visit 1 and 2; for example, an aggregate number of 4 at Visit 1 and Visit 2

### 2.3. Statistical Analyses

Continuous study variables were summarized with medians and ranges (minimum/maximum) while categorical study variables were presented as counts and percentages [18]. Data were inspected for completeness and outliers before analyses. A complete-case analysis was used to analyze variables with missing data. Statistical significance was evaluated using T-tests to calculate *p*-values between proportions, with alpha < 0.05. All analyses were carried out using an SPSS-based analytics tool.

## 3. Results

The initial number of HCPs included in the study was 61; however, nine were excluded due to the non-submission of their follow-up visit data from any of their patients. The final number of HCPs who had their charts and data analyzed for the study was 52: 87% were general pediatrics, and 12% were pediatric subspecialists (consisting of 11 pediatric allergists and five pediatric gastroenterologists).

**Figure 1:**
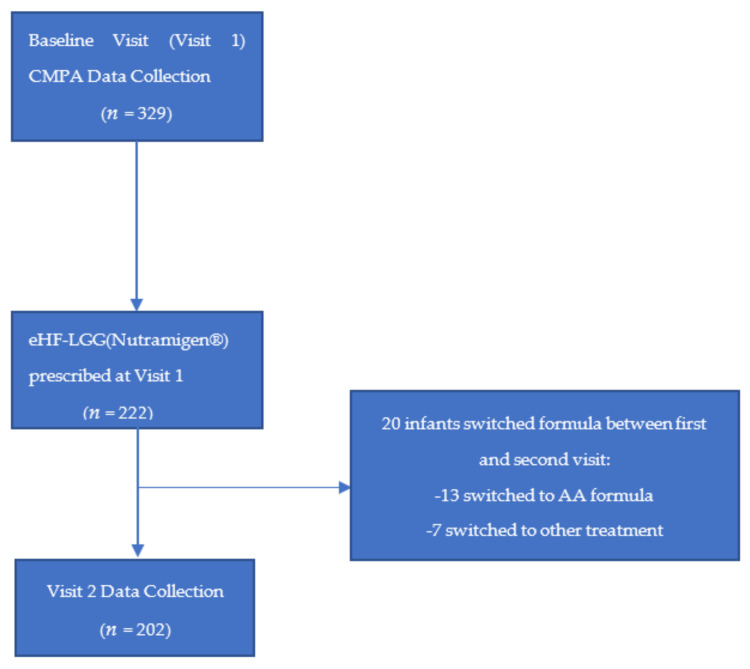
Flow Diagram of initial population vs. final population included in data analysis after application of the inclusion and exclusion study criteria.

Healthcare providers collected the data from 329 infants with suspected CMPA. Of the 329 patients, 222 infants were identified to have been placed on eHF LGG (Nutramigen^®^ Mead Johnson Nutrition, Evansville, Indiana) as their hypoallergenic formula of choice at Visit 1. Of the 222 infants placed on eHF LGG, 194 were seen by General Pediatricians (87%), with the other 28 being seen by either Pediatric GI, Pediatric A/I, or General GI (13%). Out of the 222 infants, 202 were included in the data collection at Visit 2. Figure 1 depicts the exclusion criteria being applied to the original 329 patients that had data collected at Visit 1 and Visit 2. The final study population included 202 infants who remained on the eHF LGG formula until Visit 2, as well as had data collected at both Visit 1 and Visit 2.

Patient characteristics collected from Visit 1 and Visit 2, as well as overall HCP characteristics, are depicted in Table 1. The mean age of patients at Visit 1 was 2.9 months old, with the mean age at Visit 2 being four months old. In total, 94% of the population was diagnosed via clinical assessment, with 44% of the cohort being prescribed an elimination diet as a diagnostic measure. Fifty-one percent had a family history of atopy, and 73% of the cohort had their follow-up visit at 5–6 weeks.

**Figure 2:**
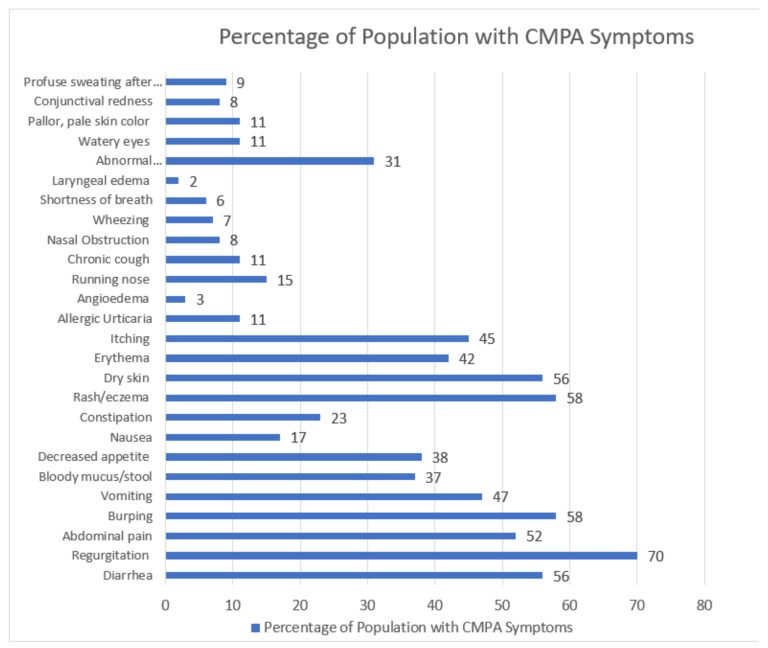
Percentage of infants with a recorded variable symptom at Visit 1 in the eHF population (*n* = 202).

Symptoms were grouped into GI, Respiratory, Skin, and Other categories. Figure 2 depicts the percentage of infants with specific CMPA symptoms at Visit 1 documented by the HCP. The most common symptoms were regurgitation (*n* = 142), rash/eczema (*n* = 117), burping (*n* = 118), and diarrhea (*n* = 117). GI and skin manifestations overall occurred in a higher percentage of patients when compared to respiratory and other symptoms.

The changes in symptoms collected via ZSMoments were then analyzed at Visit 2 following the initiation of the study formula. Using the outcome variable criteria applied to the symptom severity scale used at the initial visit, there was a reported improvement in 93% of GI symptoms (*n* = 195), 83% of skin symptoms (*n* = 147), 73% improvement of respiratory symptoms (*n* = 56), and 90 % of other symptoms (*n* = 164). The following tables depict the improvement for all analyzed symptoms: Table 2 depicts GI symptoms, Table 3 depicts skin symptoms, Table 4 depicts respiratory symptoms, and Table 5 depicts other symptoms.

**Figure 3:**
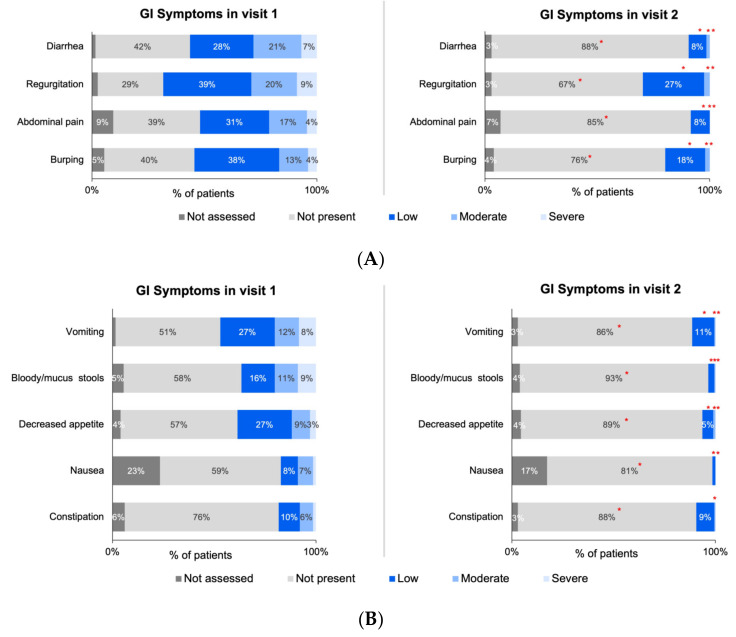
(**A**,**B**) Distribution of GI symptoms severity across initial and follow-up visits for infants ≤ 6 months of age with suspected or diagnosed CMPA. Note: ‘*’ denotes statistically significant symptom improvement between Visit 1 and Visit 2 (specific *p*-values listed in Figure S1).

**Figure 4:**
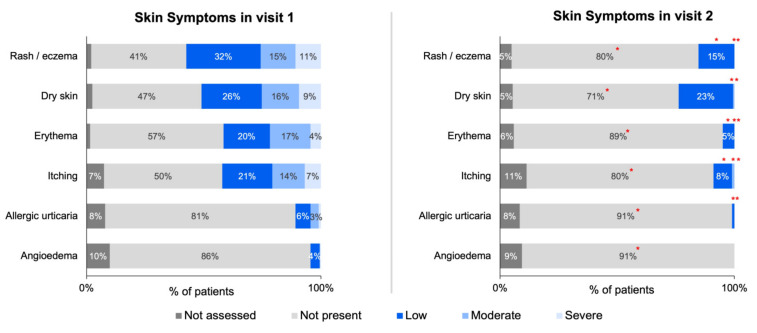
Distribution of skin symptoms severity across initial and follow-up visits for infants ≤ 6 months of age with suspected or diagnosed CMPA. Note: ‘*’ denotes statistically significant symptom improvement between Visit 1 and Visit 2 (specific *p*-values listed in Figure S2).

**Figure 5:**
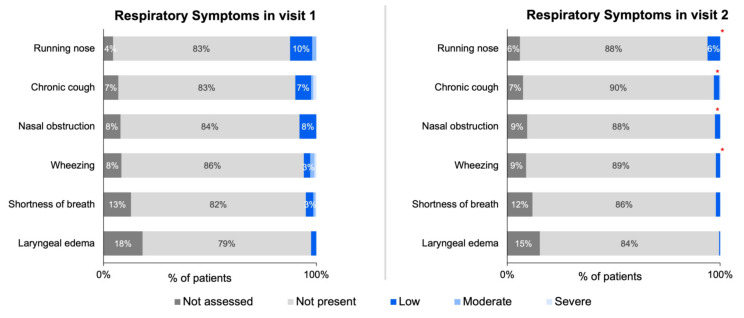
Distribution of respiratory symptoms severity across initial and follow-up visits for infants ≤ 6 months of age with suspected or diagnosed CMPA. Note: ‘*’ denotes statistically significant symptom improvement between Visit 1 and Visit 2 (specific *p*-values listed in Figure S3).

**Figure 6:**
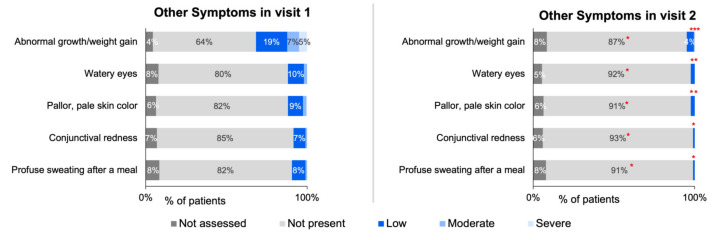
Distribution of other symptoms severity across initial and follow-up visits for infants ≤ 6 months of age with suspected or diagnosed CMPA. Note: ‘*’ denotes statistically significant symptom improvement between Visit 1 and Visit 2 (specific *p*-values listed in Figure S4).

The below figures represent the specific changes on the system severity scale regarding the 27 total symptoms that were distributed into four different categories. Figure 3A,B depicts the GI symptoms analyzed, Figure 4 depicts skin symptoms, Figure 5 depicts respiratory symptoms, and Figure 6 depicts other symptoms. The results depicted a statistically significant improvement in all GI, skin, and other symptoms when comparing the severity scale score between the first and second visit. The respiratory symptoms yielded a statistically significant improvement in the chronic cough and nasal obstruction symptoms only. The smaller sample size was noted for symptoms of allergic urticaria, angioedema, chronic cough, nasal obstruction, wheezing, shortness of breath, laryngeal edema, watery eyes, pallor, conjunctival redness, and sweating after a meal (<30 sample size).

## 4. Discussion

This prospective cohort analysis survey of de-identified clinical and demographic data analyzed infants with symptoms consistent with or diagnosed with CMPA. Utilizing eHF LGG as the hypoallergenic formula of choice, the results between Visit 1 and 2 depicted an overall reduction in severity among all four symptom categories and a statistically significant decrease regarding the presence of symptoms in the GI, skin, and other symptom categories. Using the ZSMoments system to document real-time data accurately and freely for each patient, HCPs were able to not only assess symptom improvement in the actual clinical setting but also allowed for this study to determine the short-term efficacy of eHF LGG for CMPA symptom relief.

Host and Halken summarized the diagnostic criteria for CMPA in 2014 that includes the following: elimination diet that should result in resolution of symptoms, recurrence of exact same symptoms with an oral challenge test, and lastly, ensuring that other causes of lactose intolerance and GI infections are ruled out [8]. However, due to the different onset and type of symptoms between the IgE-mediated CMPA (immediate hypersensitivity) and non-IgE-mediated CMPA (delayed hypersensitivity), it can be difficult to diagnose CMPA as there is no specific diagnostic laboratory test for non-IgE-mediated CMPA, among other reasons [1]. Clinical tools have been developed to assist in the recognition and assessment of CMPA by healthcare providers in practice, though these remain insufficient for standalone diagnosis [19]. In this study, 74% of the infants (*n* = 150) were newly diagnosed at Visit 1, with 93% of the study population being diagnosed by clinical assessment (*n* = 188). While 50% of the cohort utilized the elimination diet (*n* = 101) as a diagnostic measure, only 2% of the infants (*n* = 4) utilized the oral challenge test. While this combination is the gold standard for diagnosing both types of CMPA, the oral food challenge is commonly utilized at 9–12 months of age, which is three months older than the inclusion criteria age of our cohort [20]. Furthermore, the usage of the food challenge test is also impacted by families’ reluctance to have a re-emergence of symptoms, especially if done after an elimination diet [10]. Overall, CMPA can be difficult to diagnose due to a multitude of reasons, particularly the multi-system impact it has as lack of good biomarkers, laboratory values, or skin prick testing for non-IgE-mediated hypersensitivity [1]. Prior studies identified differences in microbiological gut markers in infants with CMPA, suggesting that development of the gut microbiome may be important in development of CMPA [21,22]. Further, the gut microbiome has been shown to change following management with the probiotic LGG [23]. Misdiagnosis of CMPA is also a clinical issue due to an unnecessary elimination diet that may lead to nutritional imbalances, increasing the risk of decreased bone mineralization and poor growth, among others [2,24]. Studies have found that the rate of reported CMPA by parents/guardians is four times higher than the actual CMPA diagnostic rate [2]. Clinical assessment and family history, which is a known risk factor of CMPA and present in 49% of this study’s infants (*n* = 100), are therefore important diagnostic tools for CMPA.

Treatment of CMPA with breastfeeding involves the mother following an elimination diet of 3–5 days if the patient has immediate CMPA symptoms and 2–3 weeks if late symptoms are present [1,25]. The second-line treatment uses hypoallergenic formula, specifically either eHF or AA formula. According to the American Academy of Pediatrics, eHF is generally preferred over AA formula, except for infants who have severe reactions, enteropathy, or those with severe growth failure or anemia [1]. In general, ESPGHAN guidelines recommend eHF due to it being more affordable and less occurrence of adverse reactions [9]. This study’s formula, eHF, had the added probiotic Lactobacillus rhamnosus GG, which has been proven to be effective in CMPA treatment [12]. The short-term efficacy of eHF-LGG for symptom improvement at the next follow-up visit has been conducted in Poland and France; however, this is the first study to our current knowledge addressing this topic in the United States [4]. Furthermore, in this survey, many symptoms were analyzed, and divided into four unique categories encompassing 27 different symptoms. To our knowledge, this is the largest number of symptoms that have been analyzed in a study testing eHF-LGG treatment formula on a study population. Overall, the aggregated improvement for each CMPA symptom division between the two visits was 93% for GI, 83% for skin, 73% for respiratory, and 90% for the other category. Analyzing the ZSMoments survey severity scale showed that every symptom in the GI and skin categories had a statistically significant increase improving to “Not Present” at Visit 2. When analyzing the respiratory category, two of the symptoms (chronic cough and nasal obstruction) had a statistically significant improvement to the “Low” severity on the scale between Visit 1 and Visit 2. Lastly, all the symptoms in the other category had a statistically significant improvement to either “Not Present” or “Low” at Visit 2 compared to Visit 1. Overall, the results of this study suggest that eHF-LGG provides clinical relief and symptom improvement in infants ≤ 6 months of age with suspected CMPA, with improvement most often by the next follow-up visit.

There are some limitations to this study. Firstly, this study lacked a control group, which limited the ability to determine if symptoms improved spontaneously or were due to treatment formula intervention. There was a small sample size of patients experiencing other (non-GI, no-skin, non-respiratory) symptoms, including allergic urticaria, angioedema, chronic cough, nasal obstruction, wheezing, shortness of breath, laryngeal edema, watery eyes, pallor, conjunctival redness, and sweating after a meal. The short-term focus of this investigation included the first patient follow-up visit (Visit 2), which limited the ability to confirm the long-term resolution of symptoms after eventual treatment cessation. Lastly, patient selection and treatment were initiated by clinician diagnosis in the outpatient setting, and not all infants underwent formal elimination rechallenge or select-IgE testing in this short-term observational period.

## 5. Conclusions

This is the largest prospective survey to occur in the US to date examining the short-term change of allergic symptoms in infants ≤ 6 months of age with suspected CMPA treated with eHF with LGG. Analysis of this prospective cohort showed improvement in all symptom categories at Visit 2 in infants ≤ 6 months of age when treated with eHF-LGG. Our study suggests that eHF-LGG may decrease symptom severity in infants ≤ 6 months of age with suspected or diagnosed CMPA, often by the next follow-up visit (Visit 2). Further randomized controlled trials are needed to confirm these preliminary results.

## Figures and Tables

**Table 1:** Patient and HCP characteristics.

Item	Population
**Demographics**	
Male	*n* = 99 (49%)
Female	*n* = 103 (51%)
Age at enrollment (months)	
● Median	3
● Mean	2.9
Time Between First and Second Visit	
● 3 weeks	*n* = 34 (17%)
● 4 weeks	*n* = 22 (10%)
● 5 Weeks	*n* = 70 (35%)
● >5 weeks	*n* = 76 (38%)
Family Allergy History	*n* = 113 (51%)
**CMPA Diagnosis Parameters**	
Time of Diagnosis	
● Newly Diagnosed	*n* = 150 (74%)
● Already Under Treatment	*n* = 72 (26%)
Method of Diagnosis	
● Clinical Assessment	*n* = 190 (94%)
● Elimination Diet	*n* = 89 (44%)
● Family History	*n* = 99 (49%)
● Skin Prick Test	*n* = 8 (4%)
● Food Panel Tests	*n* = 10 (5%)
● sIgE assay/ABMK allergen component assay	*n* = 6 (3%)
● Atopic Patch Test	*n* = 6 (3%)
● Food antigen rechallenge	*n* = 4 (2%)
**CMPA Management Strategies**	
eHF LGG (Nutramigen^®^)	*n* = 202 (100%)
Probiotics	*n* = 102 (50%)
Breastfeeding	*n* = 51 (25%)
Antihistamines	*n* = 54 (27%)
**HCP Demographics**	
Primary Specialty	
● General Pediatrics	*n* = 45 (87%)
● Pediatric GI	*n* = 3 (6%)
● Pediatric A/I	*n* = 3 (6%)
● General GI	*n* = 1 (2%)
Patient Volume (Patients/week)	
● 2–4	*n* = 22 (42%)
● 5–10	*n* = 20 (38%)
● 11–20	*n* = 9 (17%)
● >20	*n* = 1 (2%)

**Table 2:** Distribution of GI symptoms in infants ≤ 6 months of age with diagnosed or suspected CMPA.

Symptom (N ^1^)	# Patient Symptom Improvement (%) ^2^
Diarrhea (*n* = 117)	105 (90%)
Regurgitation (*n* = 142)	105 (74%)
Abdominal pain (*n* = 105)	95 (90%)
Belching/Burping (*n* = 118)	94 (80%)
Vomiting (*n* = 97)	83 (86%)
Bloody mucoid/stool (*n* = 74)	70 (95%)
Decreased appetite (*n* = 76)	69 (91%)
Nausea (*n* = 35)	34 (97%)
Constipation (*n* = 46)	31 (67%)

^1^ Number of patients who presented with a symptom at the first visit and were assessed at the second visit; ^2^ Only patients with a symptom present at the first visit were included in the percent improvement calculation.

**Table 3:** Distribution of skin symptoms in infants ≤ 6 months of age with diagnosed or suspected CMPA.

Symptom (N ^1^)	# Patient Symptom Improvement (%) ^2^
Rash/eczema (*n* = 117)	101 (86%)
Dry Skin (*n* = 113)	86 (76%)
Erythema (*n* = 85)	80 (94%)
Itching (*n* = 90)	76 (84%)
Allergic urticaria (*n* = 23)	21 (91%) *
Angioedema (*n* = 7)	7 (100%) *

^1^ Number of patients who presented with a symptom at the first visit and were assessed at the second visit; ^2^ Only patients with a symptom present at the first visit were included in the percent improvement calculation. * *n* < 30.

**Table 4:** Distribution of respiratory symptoms in infants ≤ months of age with diagnosed or suspected CMPA.

Symptom (N ^1^)	# Patient Symptom Improvement (%) ^2^
Running nose (*n* = 30)	20 (67%)
Chronic cough (*n* = 23)	19 (83%) *
Nasal Obstruction (*n* = 17)	14 (82%) *
Wheezing (*n* = 15)	11 (73%) *
Shortness of breath (*n* = 12)	8 (67%) *
Laryngeal edema (*n* = 4)	4 (100%) *

^1^ Number of patients who presented with a symptom at the first visit AND were assessed at the second visit; ^2^ Only patients with a symptom present at the first visit were included in the percent improvement calculation. * *n* < 30.

**Table 5:** Distribution of other symptoms in infants ≤ 6 months of age with diagnosed or suspected CMPA.

Symptom (N ^1^)	# Patient Symptom Improvement (%) ^2^
Abnormal growth/weight gain (*n* = 62)	56 (90%)
Watery Eyes (*n* = 23)	21 (91%) *
Pallor, pale color skin (*n* = 23)	18 (78%) *
Conjunctival redness (*n* = 16)	16 (100%) *
Sweating after meal (*n* = 18)	16 (89%) *

^1^ Number of patients who presented with a symptom at the first visit AND were assessed at the second visit; ^2^ Only patients with a symptom present at the first visit were included in the percent improvement calculation. * *n* < 30.

## Data Availability

The datasets generated and/or analyzed during the current study are not publicly available due to subject confidentiality but are available from the corresponding author on reasonable request.

## Supplementary Materials

The following supporting information can be downloaded at: https://www.mdpi.com/article/10.3390/nu15071677/s1, Figure S1. Visit 1 to Visit 2 improvement in severity of gastrointestinal symptoms for infants ≤ 6 months of age with diagnosed or suspected CMPA. Figure S2. Visit 1 to Visit 2 improvement in the severity of skin symptoms for infants ≤6 months of age with diagnosed or suspected CMPA. Figure S3. Visit 1 to Visit 2 improvement in the severity of respiratory symptoms for infants ≤6 months of age with diagnosed or suspected CMPA. Figure S4. Visit 1 to Visit 2 improvement in the severity of other symptoms for infants ≤6 months of age with diagnosed or suspected CMPA.
