# Supplementary material for: Extensively Hydrolyzed Formula Improves Allergic Symptoms in the Short Term in Infants with Suspected Cow’s Milk Protein Allergy
Source: Nutrients. 2023 Mar 30;15(7):1677. doi: 10.3390/nu15071677 (PMC10096968; doi:10.3390/nu15071677)
Supplement: Supplementary file 1 [file nutrients-15-01677-s001.zip › nutrients-2272609-supplementary.pdf]

Supplemental Figures: Symptom improvement p values

Nutramigen	Not assessed	Not present	Low	Moderate	Severe
Burping	0.482	0.000	0.000	0.000	0.004
Abdominal pain	0.365	0.000	0.000	0.000	0.002
Regurgitation	0.761	0.000	0.011	0.000	0.000
Diarrhea	0.313	0.000	0.000	0.000	0.000
Constipation	0.149	0.002	0.614	0.001	0.082
Nausea	0.138	0.000	0.001	0.000	0.082
Decreased appetite	0.805	0.000	0.000	0.000	0.014
Bloody/mucus stools	0.482	0.000	0.000	0.000	0.000
Vomiting	0.313	0.000	0.000	0.000	0.000

Figure S1. P-values for Visit 1 and 2 symptom improvement for gastrointestinal symptoms for infants <u><</u>6 months of age with diagnosed or suspected CMPA. Note: '-' denotes undefined p-values (null entries across visits).

Nutramigen	Not assessed	Not present	Low	Moderate	Severe
Angioedema	0.867	0.125	0.004	-	0.318
Allergic urticaria	0.856	0.007	0.004	0.008	0.157
Itching	0.174	0.000	0.000	0.000	0.000
Erythema	0.018	0.000	0.000	0.000	0.002
Dry skin	0.126	0.000	0.564	0.000	0.000
Rash / eczema	0.103	0.000	0.000	0.000	0.000

Figure S2. P-values for Visit 1 and 2 symptom improvement for skin symptoms for infants ≤6 months of age with diagnosed or suspected CMPA. Note: '-' denotes undefined p-values (null entries across visits).

Nutramigen	Not assessed	Not present	Low	Moderate	Severe
Laryngeal edema	0.426	0.199	0.100	-	-
Shortness of breath	0.763	0.277	0.360	0.157	0.318
Wheezing	0.860	0.296	0.523	0.045	0.157
Nasal obstruction	0.597	0.250	0.014	-	-
Chronic cough	0.848	0.060	0.022	0.563	0.082
Running nose	0.503	0.157	0.103	0.045	-

Figure S3. P-values for Visit 1 and 2 symptom improvement for respiratory symptoms for infants ≤6 months of age with diagnosed or suspected CMPA. Note: '-' denotes undefined p-values (null entries across visits).

Nutramigen	Not assessed	Not present	Low	Moderate	Severe
Profuse sweating after a meal	0.856	0.008	0.000	0.157	-
Conjunctival redness	0.686	0.007	0.001	0.157	-
Pallor, pale skin color	1.000	0.006	0.003	0.045	0.318
Watery eyes	0.320	0.001	0.002	0.045	-
Abnormal growth/weight gain	0.105	0.000	0.000	0.000	0.001

Figure S4. P-values for Visit 1 and 2 symptom improvement for other symptoms for infants ≤6 months of age with diagnosed or suspected CMPA. Note: '-' denotes undefined p-values (null entries across visits).
